# Supplementary figures and images for: Stochastic Processes Derive Gut Fungi Community Assembly of Plateau Pikas (Ochotona curzoniae) along Altitudinal Gradients across Warm and Cold Seasons
Source: J Fungi (Basel). 2023 Oct 20;9(10):1032. doi: 10.3390/jof9101032 (PMC10607853; doi:10.3390/jof9101032)

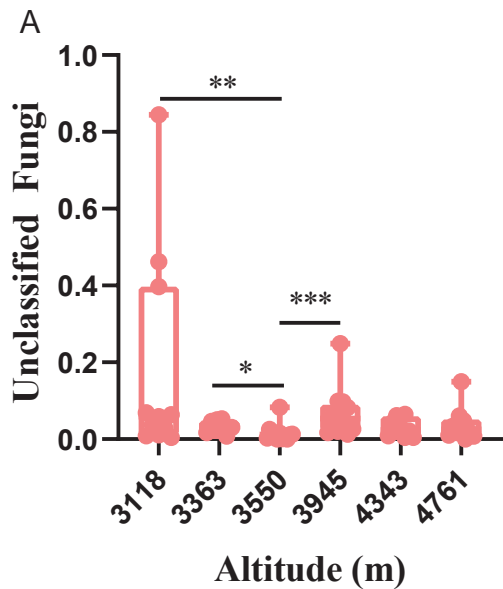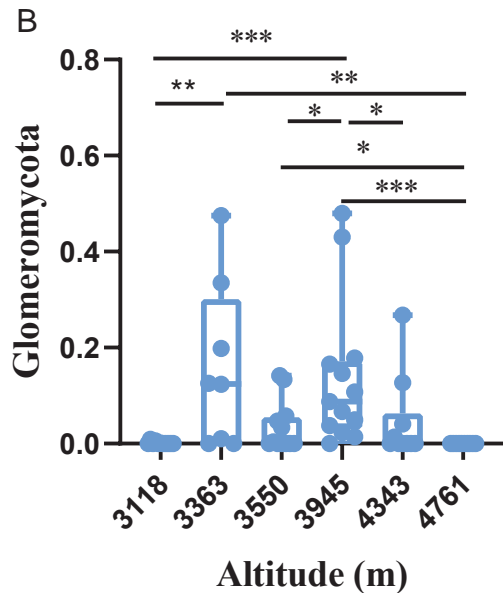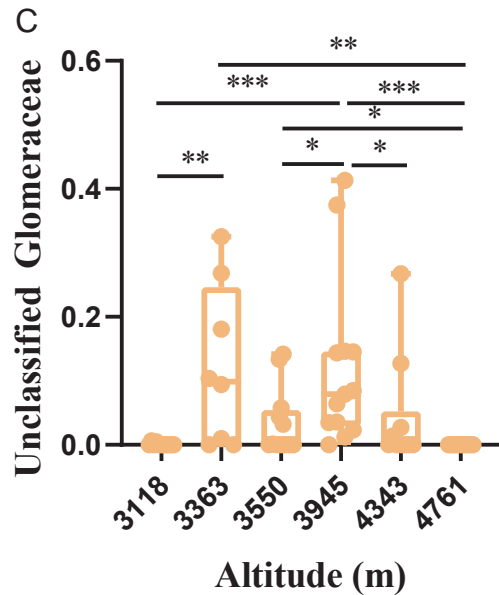

Supplement: Supplementary file 1 [file jof-09-01032-s001.zip › Fig.S1.pdf]

A

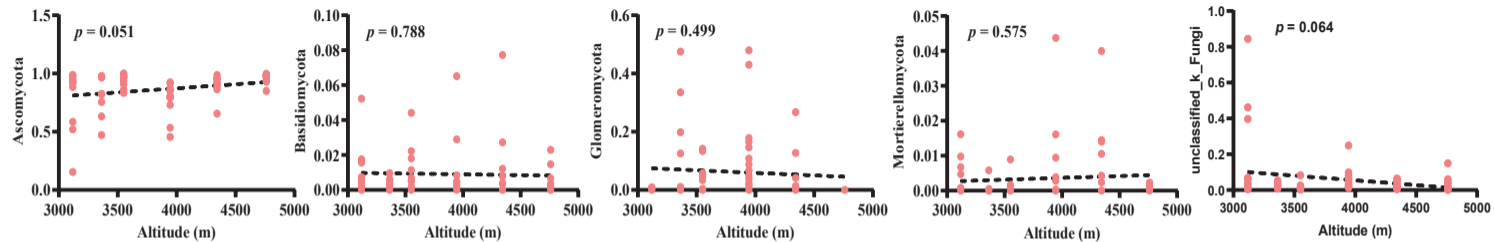

B

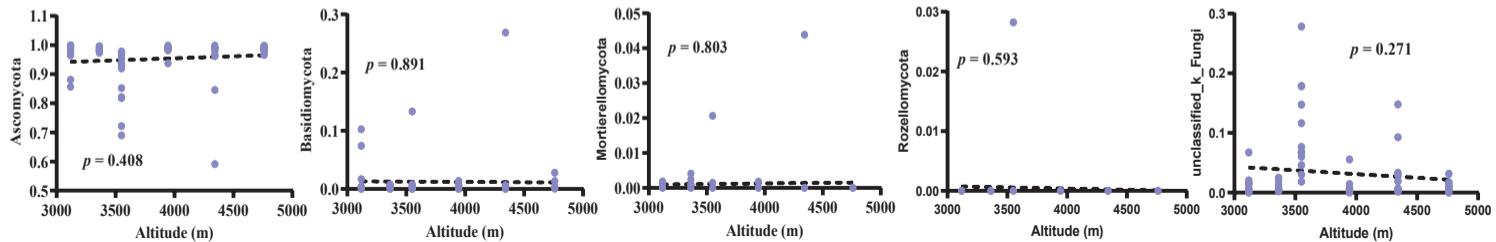

Supplement: Supplementary file 1 [file jof-09-01032-s001.zip › Fig.S2.pdf]

**A**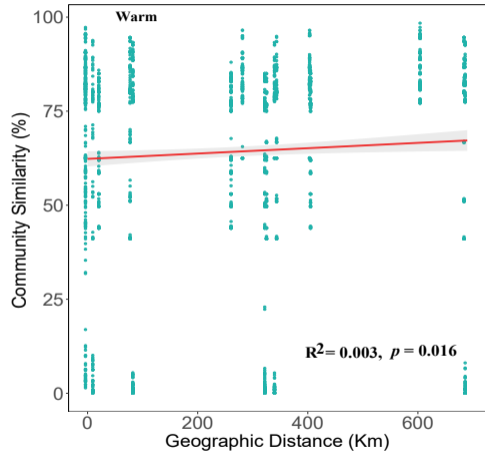**B**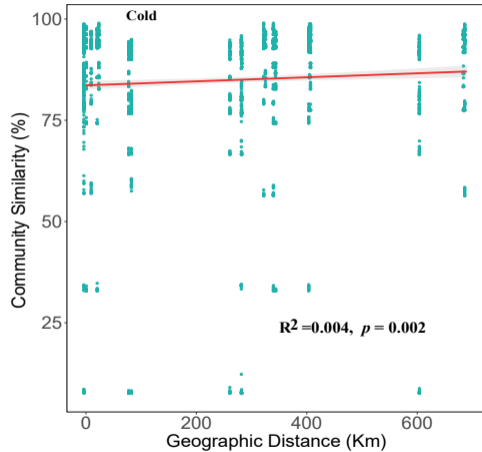**C**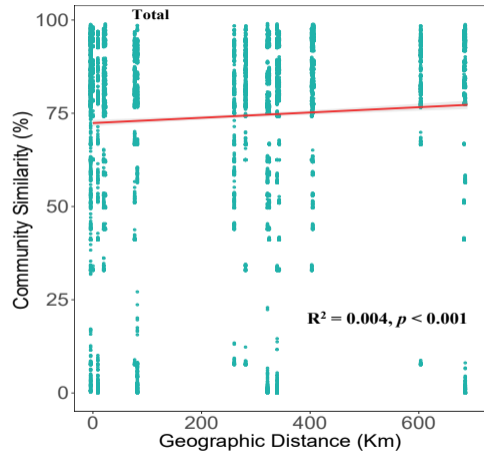

Supplement: Supplementary file 1 [file jof-09-01032-s001.zip › Fig.S3.pdf]
